# ART for prevention: opportunities, challenges and predictions

**DOI:** 10.1186/1471-2334-14-S2-S7

**Published:** 2014-05-23

**Authors:** Myron S Cohen

**Affiliations:** 1 University of North Carolina, Chapel Hill, North Carolina, 28443, USA

## 

Many antiretroviral agents (ARVs) concentrate in genital secretions. When provided to people with HIV these agents stop the transmission of the virus, presumably by i) reducing the concentration of virus and ii) bathing vulnerable tissues with ARVs during the potential transmission. In 11/13 observational studies ART reduced HIV transmission. In one observational study that included gay couples, treatment of the infected partner stopped transmission. In one randomized controlled trial (HPTN 052) of 17623 couples at 13 sites in 9 countries, documented suppression of viremia appears to have been 100% effective in prevention of HIV transmission. The maximal potential of ARVs for prevention of HIV requires universal early or immediate treatment, with good adherence to therapy. Currently only a few countries (including the US and Brazil) have guidelines leading to immediate treatment of infection. In addition, wider testing is required to identify HIV infected people who do not know their status. And new tests must be developed to detect acute HIV infection, a short stage of disease that contributes disproportionately to the spread of HIV. Earlier ART benefits the health of the HIV infected person: CD4 cells are restored to a high level, tuberculosis is averted, subtle changes in mentation are avoided, and normal lifespan is restored. While the biomedical benefits of early treatment of HIV have been established, logistical challenges of treatment-including early treatment-are considerable; and such logistical challenges compromise more immediate utilization of ARVs. It can be anticipated that universal immediate ART will become the standard of care in the near future. Commitment to simplifying treatment regimens and improving infrastructure is essential. In addition, HIV infected patients must be inspired to protect their own health through treatment. Ongoing confusion about when to start ART among patients and providers convinces asymptomatic people with HIV infection that delay of treatment has no adverse consequence, which does not appear to be the case in either short or long time frames. While treatment can be difficult, people who are not offered ARVs are often lost to follow-up. ARVs represent the cornerstone of combination HIV prevention; optimizing the clinical and public health benefit(s) of these agents is of the greatest priority.

